# Patient Sex and Physician Gender as Modifiers of Low-density Lipoprotein Cholesterol Control in High-risk Patients of Atherosclerotic Disease: A Cross-sectional Study

**DOI:** 10.31662/jmaj.2024-0245

**Published:** 2024-12-20

**Authors:** Hiromitsu Yamashita, Nozomi Kubota, Masayoshi Shiota

**Affiliations:** 1 Kawakita Family Clinic Minamiasagaya, Tokyo, Japan

**Keywords:** Gender concordance, LDL cholesterol, Primary and secondary prevention, Atherosclerotic cardiovascular disease, Evidence-practice gap

## Abstract

**Introduction:**

Inadequate management of low-density lipoprotein (LDL) cholesterol is more common in female patients than in male patients in the context of preventing atherosclerotic cardiovascular disease. Moreover, the effect of physician gender on patient outcomes has been acknowledged. However, to date, no study in Japan has investigated this issue or explored the potential interactions between patient sex and physician gender. This study aimed to assess disparities in achieving LDL cholesterol targets between male and female patients and examine the impact of the patient-physician gender dyad.

**Methods:**

We conducted a cross-sectional study using electronic medical records from an urban Japanese clinic. Patients aged 40-79 years with coronary artery disease, noncardiogenic stroke, or diabetes mellitus were included in the study. The modified Poisson regression model with robust error variance was used, and patients were stratified by sex to evaluate the interaction between patient sex and physician gender.

**Results:**

Among the 714 patients (44.1% women), female patients were less likely to achieve LDL cholesterol targets than male patients (70.7% male vs. 63.9% female). Adjusted analyses revealed that this trend persisted for female patients (adjusted prevalence ratio: 0.86, 95% confidence interval [CI]: 0.77-0.96). A notable interaction between patient sex and physician gender was observed; male patients managed by female physicians had lower LDL cholesterol target achievement than male patients managed by male physicians (adjusted prevalence ratio: 0.74 [95% CI: 0.62-0.88]).

**Conclusions:**

Female patients were less likely to achieve LDL cholesterol targets, and patient-physician gender discordance was associated with poorer lipid management. These findings highlight the need for quality improvement interventions to address the disparity.

## Introduction

For the primary and secondary prevention of atherosclerotic diseases, comprehensive evaluation, and management of risk factors, such as dyslipidemia, hypertension, diabetes mellitus, and smoking, are recommended ^(1), (2)^. The Japanese guideline ^(2)^ suggests calculating an absolute risk score for the development of atherosclerotic diseases in each patient and setting low-density lipoprotein (LDL) cholesterol targets according to the degree of risk. Specifically, for secondary prevention, a target LDL cholesterol level <100 mg/dL is recommended for patients with a history of coronary artery disease, whereas LDL cholesterol level <120 mg/dL is recommended for those with a history of noncardiogenic stroke, regardless of the patient’s sex ^(2)^. For the primary prevention, a target LDL cholesterol level <120 mg/dL is also recommended for patients with a history of diabetes mellitus, regardless of the patient’s sex.

Recent studies have emphasized how sex and gender interact as modifiers in health-related matters, including disease prevalence, treatment response, and health-seeking behaviors ^(3)^. The term “sex” generally refers to the biological, sexual, and reproductive distinctions between male and female, whereas “gender” generally refers to the social norms, perceptions, and roles associated with being a woman or a man ^(3)^. In the context of atherosclerotic disease prevention, previous studies have reported that LDL cholesterol is less likely to be optimally managed in female than in male patients ^(4), (5), (6), (7), (8), (9), (10), (11)^. In addition to patient sex, healthcare provider attributes, including gender, may contribute to various patient outcomes. For example, a study focusing on diabetes mellitus reported that male patients were less likely to have adequately controlled LDL cholesterol level, particularly when managed by female primary care physicians ^(12)^. Other studies also demonstrated interactions between the patient’s and the physician’s gender in various healthcare settings, including emergency and surgical settings ^(13), (14), (15), (16)^.

The interaction between patient sex and physician gender may have important implications for personalized healthcare and health equity. However, no study has yet investigated this dynamic in high-risk patients, including those with a history of atherosclerotic disease, in a primary care setting. Furthermore, although a previous study conducted in Japan has focused on primary prevention ^(11)^, no study has determined whether LDL cholesterol is not adequately controlled in female patients requiring secondary prevention. Therefore, the present study aimed to test the hypothesis that male and female patients differ in achieving LDL cholesterol targets for the prevention of atherosclerotic disease in a primary care setting. In addition, we investigated the potential interaction between patient sex and physician gender. The findings of this study could contribute to informing clinical practices and ensuring more effective and equitable healthcare delivery, thereby advancing personalized healthcare and addressing health equity concerns.

## Materials and Methods

### 1 Study design and data source

This was a cross-sectional study conducted at a primary care-providing urban clinic in Tokyo, which does not have inpatient beds. The clinic accommodates approximately 120 outpatients daily. During the study period, a total of 22 physicians provided outpatient care in the clinic.

### 2 Participants

This study included patients aged 40-79 years, with a documented history of coronary artery disease, noncardiogenic stroke (i.e., atherothrombotic stroke or lacunar infarction), or diabetes mellitus, who underwent followup examinations between April 1, 2022, and June 30, 2022. In Japan, patients with chronic diseases visit their clinic at least once every 3 months to ensure that all eligible participants were included during the study period.

The following exclusion criteria were established:

1) Patients whose first visit occurred within 6 months preceding April 1, 2022.

Considering the possibility that patients with a shorter consultation period may not have undergone adequate evaluations and interventions, such as lifestyle counseling and statin prescriptions, we excluded those who were within 6 months from their initial visit.

2) Patients with a usual provider continuity index (UPC) ≤0.5.

The UPC index was calculated as the ratio of the number of opportunities to see the primary care physician to all the opportunities for clinic visits. It serves as a measure of care continuity ^(17)^. UPC index scores are sometimes categorized into two groups, with scores higher than 0.5 considered to be high ^(18)^. We defined the primary physician in charge as “a physician with UPC >0.5 in one year,” and patients with a UPC ≤0.5 (i.e., patients without a primary care physician with a UPC > 0.5) were excluded.

### 3 Study variables

#### 3.1 Outcomes

The primary outcome was the achievement of the LDL cholesterol target. The LDL cholesterol values used for the analysis were the most recent values documented in the medical records between October 2021 and June 2022. As LDL cholesterol was not necessarily measured at each visit, the restriction of data collection to the followup period alone would have resulted in a substantial amount of missing data. To alleviate this, we extended the data extraction period by 6 months prior to the followup period to ensure more comprehensive data. The LDL cholesterol target for patients with a history of coronary artery disease was set at <100 mg/dL, whereas that for patients with a history of noncardiogenic stroke or diabetes mellitus was set at <120 mg/dL. The secondary objective was to determine whether there was an interaction between patient sex and physician gender.

#### 3.2 Patient sex and physician gender

Patient sex was determined on the basis of the sex indicated on their insurance card, either male or female. This sex is automatically classified in Japan to be the same as the sex assigned at birth. Physician gender was determined by asking each physician whether they were commonly perceived as a “man” or “woman” by patients and other medical professionals. The responses provided by the physicians were used to define them as either a man or woman. This concept of gender is particularly known as “gender relations” ^(3)^. Self-identified gender was not adopted to avoid pressuring physicians into disclosing personal information they may have preferred to keep private. Sex and gender are different terms; thus, we used these words according to their definitions in this study.

#### 3.3 Covariates

On the basis of previous studies ^(4), (5), (6), (7), (8), (9), (10)^, we used age, body mass index (BMI), and smoking history as covariates. In addition, physician gender, physician experience (postgraduate year), physician specialty, statin use, and antihypertensive drug use were included as covariates in the analysis. Age, BMI, and physician experience were used as continuous variables, whereas smoking history and physician specialty were used as categorical variables. Smoking was distinguished between current smokers and nonsmokers, including individuals who had smoked in the past and those who had never smoked. Physician specialty was categorized into three groups: general practice, other specialties (internal medicine), and trainee of the general practice. Statin use and antihypertensive drug use were used as binary variables. The most recently available data from the medical records between October 2021 and June 2022 were used for estimating BMI and smoking history as well as LDL cholesterol.

### 4 Statistical analyses

We employed *t*-tests to compare the averages of continuous variables, such as age, and chi-squared tests to compare the proportions of categorical variables, such as smoking history, between the patients’ sex. Regarding physician characteristics, we used the Fisher exact test to compare the categorical variable (i.e., physician specialty). A modified Poisson regression model with robust error variance ^(19)^ was used to evaluate the association between the achievement of LDL cholesterol targets and patient sex while adjusting for the covariates. The modified Poisson regression model is particularly suitable for outcomes with high prevalence and enables the direct calculation of prevalence ratios ^(19), (20)^. A complete case analysis was employed, including only participants with no missing data.

To explore the potential interaction between patient sex and physician gender, we conducted subgroup analyses. The participants were stratified by sex, and the modified Poisson regression model with robust error variance was used. After verifying multicollinearity, all covariates were included in the model. All statistical analyses were conducted using the Stata software (version 16.1; StataCorp LLC, College Station, TX, USA). Statistical significance was defined as a two-sided *P*-value < 0.05.

### 5 Ethical considerations

This study did not involve any interventions. Therefore, informed consent was not required. Instead, the patients were provided with an opt-out option, which was communicated through the clinic’s website. The patients were informed about the study and given the opportunity to opt-out if they did not wish to have their data included. The study protocol was approved by the Ethics Committee of the Kawakita General Hospital (approval number: 2022-0022).

## Results

### 1 Participant characteristics

**Figure 1.**
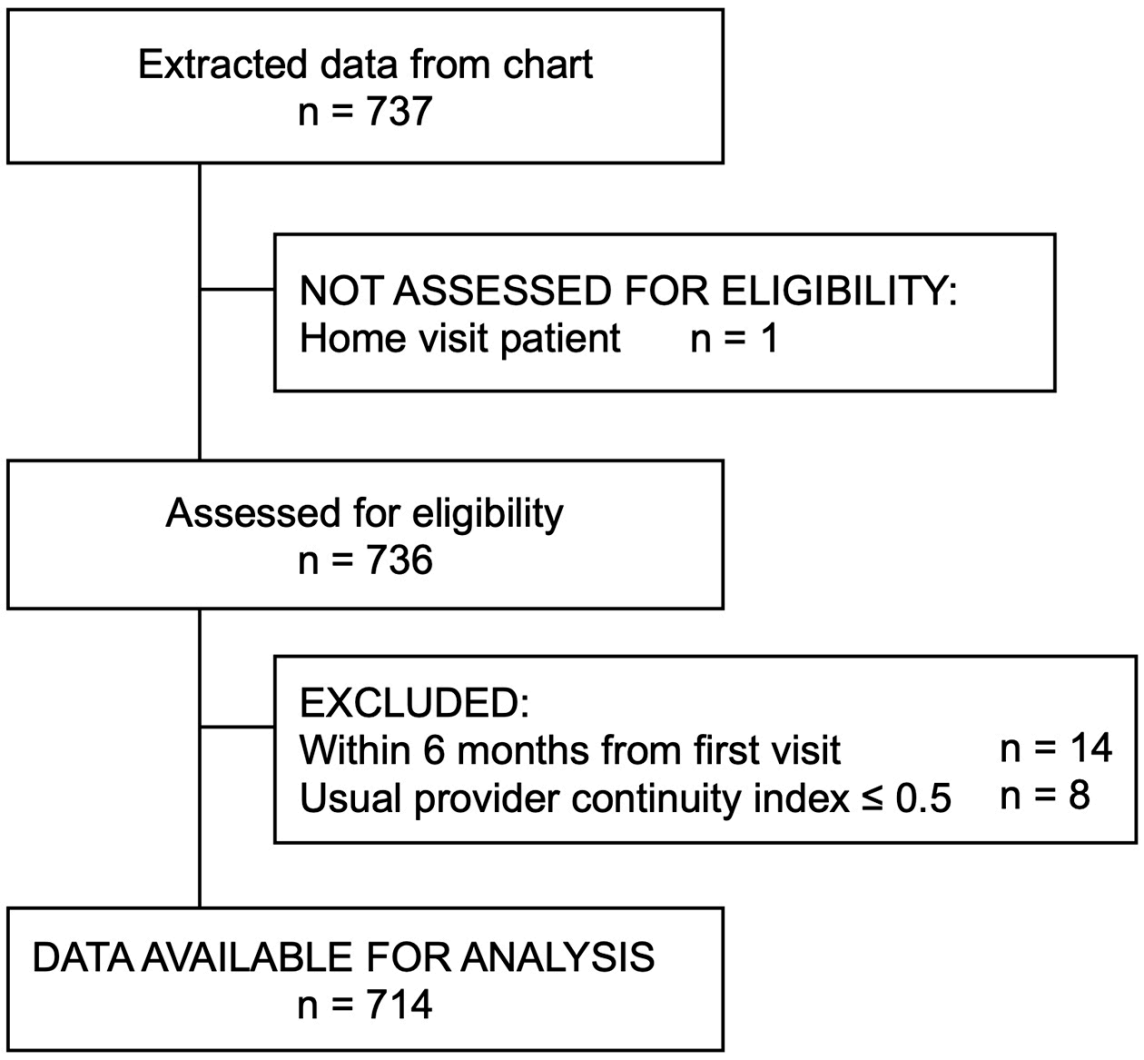
Flow chart of patient selection.

A total of 737 patients with a history of coronary artery disease, noncardiogenic stroke, or diabetes mellitus were identified through the medical record review (Figure 1). After applying the eligibility criteria, 714 individuals were included in the final analysis. Table 1 summarizes the characteristics of the study participants stratified by sex. Of the 22 physicians, 10 were women, and compared with male physicians, female physicians had a higher proportion of female patients (54.0% of all female patients). In terms of disease distribution with respect to patient sex, a higher proportion of male patients had diabetes mellitus (99.8%), whereas a higher proportion of female patients had coronary artery disease (14.0%) and stroke (36.8%). Missing data were most observed for BMI, with the proportion of missing values being 7.6%. Table 2 presents the characteristics of the physicians, showing that female physicians had longer terms of experience than male physicians (*P* = 0.014). Table 3 presents the proportion of patients who achieved LDL cholesterol targets by patient sex. Overall, a lower proportion of female patients achieved LDL cholesterol targets than male patients (70.7% of male patients vs. 63.9% of female patients). Similarly, a lower proportion of female patients achieved management targets for each disease (coronary artery disease: 85.7% of male patients vs. 67.4% of female patients; noncardiogenic stroke: 83.3% of male patients vs. 74.1% of female patients; diabetes mellitus: 69.6% of male patients vs. 58.1% of female patients).

**Table 1.** Characteristics of the Study Subjects.

Characteristics	Male (n = 399)		Female (n = 315)		Missing value, n	*P*-value
**Demographics**						
Age, years (mean [SD])	63.8	(10.3)	66.9	(9.4)	0	0.0001
Smoking					11	
Current smoker	113	(28.5%)	42	(13.7%)		<0.0001
Never smoker/past smoker	283	(71.5%)	265	(86.3%)		
BMI (mean [SD])	26.0	(4.4)	25.1	(4.5)	54	0.006
Physician’s gender					0	
Women	184	(46.1%)	170	(54.0%)		0.037
Statin use	222	(55.6%)	208	(66.0%)		0.005
Antihypertensive drug use	217	(54.4%)	164	(52.1%)		0.54
LDL cholesterol, mg/dL (mean [SD])	105.6	(27.3)	109	(26.9)	8	0.08
**Disease history**						
Coronary artery disease	28	(7.0%)	44	(14.0%)		0.002
Stroke	45	(11.3%)	116	(36.8%)		<0.0001
Atherothrombotic stroke	22	(5.5%)	33	(10.5%)		0.014
Lacuna infarct	22	(5.5%)	76	(24.1%)		<0.0001
Coexistence/indistinguishable	1	(0.3%)	7	(2.2%)		0.013
Diabetes mellitus	398	(99.8%)	178	(56.5%)		<0.0001

BMI: body mass index, LDL: low-density lipoprotein

**Table 2.** Characteristics of the Physicians.

Characteristics (Men: n = 12, Women: n = 10)	Men (n = 12)		Women (n = 10)		*P*-value
**Physician experience, years (mean [SD])**	10.5	(4.1)	26.3	(5.2)	0.014
**Specialty**					
General practice, n	7	(58.3%)	2	(20.0%)	0.06
Other specialties (internal medicine), n	2	(16.7%)	7	(70.0%)
Trainee of general practice, n	3	(25.0%)	1	(10.0%)

**Table 3.** Achievement Proportion of the LDL Cholesterol Target by Diabetes Mellitus, Coronary Artery Disease, and Noncardiogenic Stroke.

	Full sample		*P*-value	Coronary artery disease*		*P*-value	Noncardiogenic stroke^†^		*P*-value	Diabetes mellitus^‡^		*P*-value
**Patient’s sex**												
Male, n	280	(70.7%)	Ref.	24	(85.7%)	Ref.	35	(83.3%)	Ref.	256	(69.6%)	Ref.
Female, n	198	(63.9%)	0.054	29	(67.4%)	0.084	80	(74.1%)	0.23	101	(58.1%)	0.008
**Patient’s sex-physician’s gender dyad**												
Male patient × Men physician, n	160	(75.1%)	Ref.	16	(94.1%)	Ref.	26	(92.9%)	Ref.	144	(73.5%)	Ref.
Male patient × Women physician, n	120	(65.6%)	0.037	8	(72.7%)	0.11	9	(64.3%)	0.019	112	(65.1%)	0.082
Female patient × Men physician, n	89	(63.1%)	Ref.	19	(73.1%)	Ref.	34	(75.6%)	Ref.	40	(53.3%)	Ref.
Female patient × Women physician, n	109	(64.5%)	0.80	10	(58.8%)	0.33	46	(73.0%)	0.767	61	(61.6%)	0.27

Note: Patients with missing LDL cholesterol data were excluded from the analysis (3 men and 5 women were excluded). * LDL cholesterol target: <100 mg/dL † Noncardiogenic stroke is specifically atherothrombotic stroke and/or lacunar infacrion. Excluding those with coronary artery disease. LDL cholesterol target: <120 mg/dL ‡ Excluding those with coronary artery disease. LDL cholestrol target: <120 mg/dL

### 2 Primary analysis

**Figure 2.**
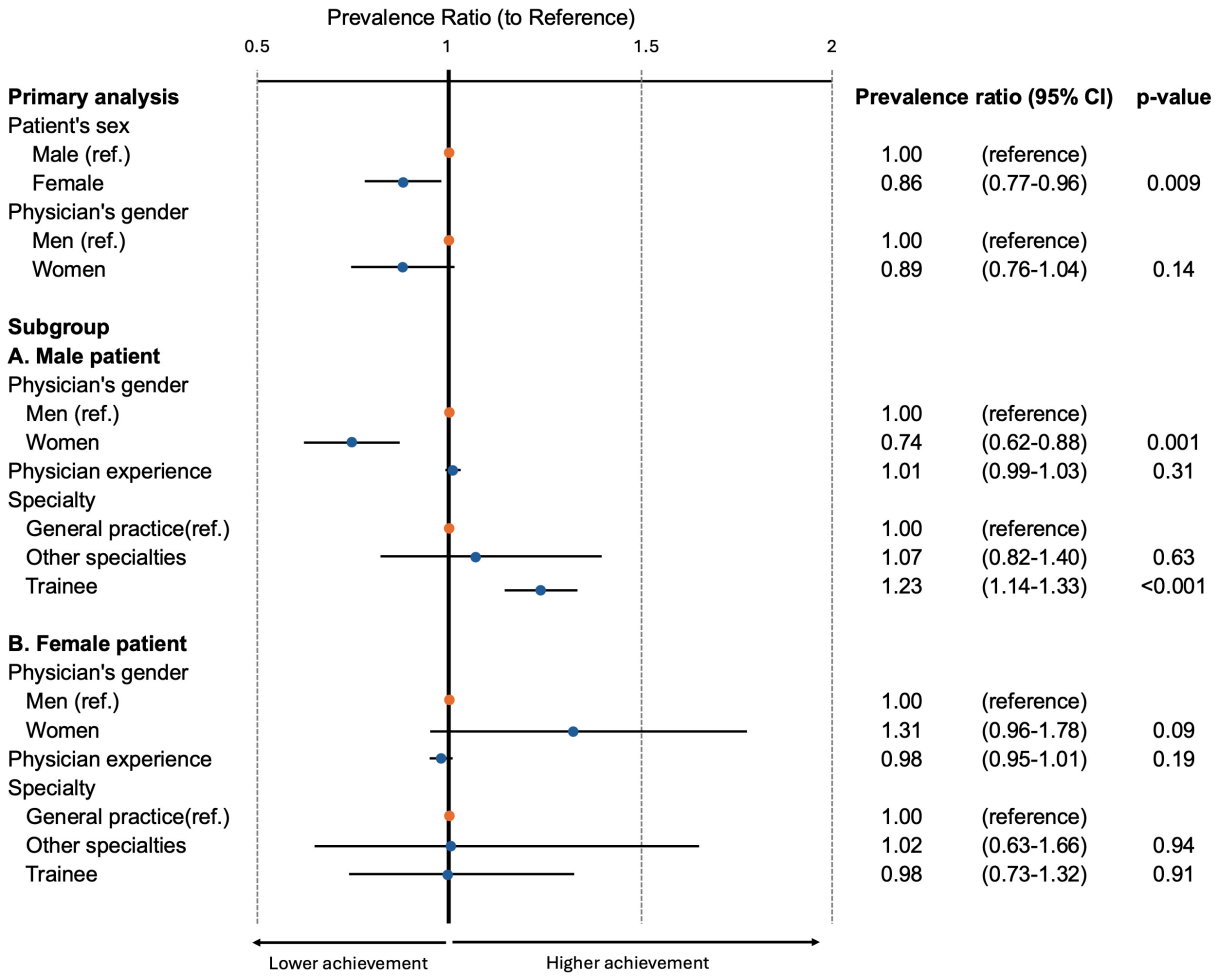
Results of regression models estimating the association between achievement of LDL cholesterol targets and patient sex LDL, low-density lipoprotein; CI, confidence interval; ref., reference.

Figure 2 illustrates the association between the achievement of LDL cholesterol targets and patient sex. The modified Poisson regression model analysis revealed that female patients had a significantly lower chance of achieving their LDL cholesterol targets than male patients (adjusted prevalence ratio [aPR] 0.86, 95% confidence interval [CI]: 0.77-0.96). In terms of physician gender, after adjusting for covariates, the difference was not significant (aPR 0.89, 95% CI: 0.76-1.04).

### 3 Secondary analyses

The results of subgroup analyses according to patient sex are also presented in Figure 2. An interaction was observed between patient sex and physician gender, with the interaction term for female physicians managing male patients yielding an aPR of 0.74 (95% CI: 0.62-0.88) compared with the reference group (male physician-male patient combination). No significant interaction was observed between female patients and male or female physicians (aPR 1.31, 95% CI: 0.96-1.78). In terms of physician specialty, trainees of general practice were associated with higher LDL cholesterol target achievement in male patients (aPR 1.23, 95%CI: 1.14-1.33), but this difference was not observed in female patients.

## Discussion

### 1 Summary of the main findings

Consistently, female patients exhibited a lower chance of achieving management targets than male patients. After adjusting for covariates, our analysis showed that female patients were less likely to achieve their LDL cholesterol control targets compared with male patients. In addition, an interaction effect between patient sex and physician gender was observed, resulting in a lower proportion of patients achieving the management target when male patients were managed by female physicians. Moreover, trainees of the general practice were associated with higher LDL cholesterol target achievement in male patients, but this was not observed in female patients.

### 2 Interpretation of the findings

Consistent with previous studies reporting a higher likelihood of suboptimal LDL cholesterol management in female patients ^(4), (5), (6), (7), (8), (9), (10)^, our findings confirmed a similar pattern in an urban clinic in Japan. Mechanisms such as lower statin therapy offer rates and higher discontinuation rates among female than male patients may underlie these disparities ^(10)^. In addition, our study provided novel insights into the patient-physician dynamic by revealing that the interaction between patient sex and physician gender influences the proportion of patients who achieve the LDL cholesterol target in the context of atherosclerotic disease prevention. These findings highlight the importance of implementing tailored interventions and strategies to address the disparity and enhance the outcomes in patients undergoing atherosclerotic disease management.

The identification of the interaction between patient sex and physician gender in relation to LDL cholesterol management adds complexity to the understanding of the patient-physician gender dynamics. A systematic review previously indicated that the combination of patient and physician sexes can influence medical practices ^(21)^. As regards the combination of male patients and female physicians, female physicians’ voice tone is least friendly, characterized by the least calmness. The review suggested tension around gender role conflict, rendering the negotiation of these consultations more difficult ^(21)^. This might explain why the combination of male patients and female physicians achieved lower target proportions than other dyads. As regards patient preferences, female patients tended to prefer consulting with female doctors ^(22)^. In our study as well, female patients tended to visit female physicians, which could reflect their preferences. As this study did not examine the mechanism of interaction between patient sex and physician gender in detail, these aspects are recommended to be investigated in future research.

In our study, compared with other specialties, trainees achieved substantially higher LDL cholesterol management targets in male patients. This difference could be attributed to receiving direct and focused supervision, which can lead to the application of the most current and evidence-based practices in patient care. This continual learning environment may enhance the trainees’ ability to meet treatment targets ^(23)^. Conversely, the lower achievement proportions observed in female patients across all physician genders and specialties indicate the influence of implicit gender biases in clinical decision-making. Implicit gender biases can shape clinical decision-making processes and subsequent treatment approaches ^(24), (25), (26)^. An example of implicit gender bias is the consideration that ischemic heart disease and chronic obstructive pulmonary disease occur in men, not in women ^(16), (27)^. In our study, patients with coronary artery disease, stroke, and diabetes mellitus consistently encompassed a lower proportion of female than male patients achieving the target LDL cholesterol level. One hypothesis is that the results may have been caused by an implicit bias, such as “LDL cholesterol is a more significant issue for males than for females.” This hypothesis would be supported by the observation that attending physicians―who guide resident training―may also harbor these biases, potentially affecting the quality of education and feedback provided to the trainees regarding female patients.

The complex nature of sex and gender necessitates further exploration as they can be defined and understood in various ways. A prospective study reported that the effective control of cardiovascular factors, such as dyslipidemia and diabetes, was more closely associated with gender than with biological sex ^(28)^. In this study ^(28)^, the authors constructed a sex index for patients based on masculine and feminine sex-related characteristics. We specifically examined the interaction between patient sex, as indicated on the insurance card (which closely aligns with the sex assigned at birth), and physician gender ^(3)^. Notably, detailed research analyzing physician gender and its association with patient sex is still lacking. Therefore, further investigation of sex and gender, encompassing both patients and physicians, would be of great importance.

### 3 Implications for educational practice

Our findings highlight the need for improved gender- and sex-specific medical education to address disparities associated with patient sex and physician gender ^(3), (13), (29)^. Currently, although quality improvement interventions for the prevention of atherosclerotic disease have been implemented, clarity regarding the adequate integration of education in sex-specific disparities is lacking ^(30), (31), (32)^. The incorporation of education that considers sex and gender in healthcare practices could help address the identified disparities in LDL cholesterol management ^(24), (26)^. Previous research has demonstrated that educational programs for healthcare providers that include explanations of clinical practice guidelines can improve the quality of care ^(33)^. Therefore, the guidelines should explicitly highlight the importance of considering sex and gender aspects ^(3)^. In doing so, healthcare providers will be better equipped to deliver tailored and effective care that considers the unique health needs of diverse genders.

### 4 Limitations

The current study has four limitations. First, it was limited in terms of external validity as it was conducted at a single center. However, the findings were consistent with those of previous studies reporting that female patients, in general, have poorer LDL cholesterol control than male patients. Moreover, the proportion of patients achieving LDL cholesterol targets in our study aligns with the findings from previous studies conducted in Japan ^(11), (34)^. Therefore, we believe that the external validity is supported to some degree. Second, this study acknowledged a limitation regarding the use of socioeconomic status as a covariate. Previous studies ^(4), (5), (6), (7), (8), (9), (10)^. included socioeconomic factors, such as educational background and income, as potential confounders. The absence of these variables in the current analysis may have resulted in residual confounding as unmeasured socioeconomic factors could have influenced the results. The decision to limit the number of explanatory variables in the study was based on the limited sample size, which could affect the statistical power and stability of the analysis. To prioritize the most relevant covariates, age, BMI, and smoking history were chosen as important factors related to LDL cholesterol management in the context of the objectives of this study. Third, it was impossible to consider diverse gender identities in the paradigm of the gender binary concept. In this study, patient sex was determined on the basis of the sex indicated on their insurance card. The analyses did not include any information on transgender individuals. As a result, misclassification of transgender people could occur (e.g., a trans woman whose sex is listed as male on the insurance card but is receiving feminizing hormones or has undergone gender-affirming surgery and changed her sex from male to female on the insurance card) ^(35)^. However, strict definition of patient sex and physician gender, as in this study, is unprecedented in the context of this topic. The inclusion of more sophisticated gender measures, such as one used in a previous study ^(28)^, could provide valuable insights for future research. Fourth, LDL cholesterol levels fluctuate over time, which could have influenced the study results. Future studies may benefit from incorporating sensitivity analyses that use the mean LDL cholesterol values over 2 or 3 measurements, rather than solely relying on single-point measurements, as in our study, to provide a more accurate assessment of lipid control.

### 5 Conclusions

The proportion of female patients achieving LDL cholesterol targets was lower than that of male patients. Furthermore, an interaction effect was observed between patient sex and physician gender, resulting in a lower proportion of patients achieving the management target, particularly when female physicians managed male patients. Educational interventions are recommended to address these disparities.

## Article Information

### Conflicts of Interest

None

### Acknowledgement

 We would like to express our gratitude to Dr. Yusuke Kanakubo for his valuable insights regarding the misclassification of transgender individuals, which helped us identify this limitation in our study.

### Author Contributions

Hiromitsu Yamashita: Conceptualization, Resources, Methodology, Software, Formal analysis, Interpretation of data, Writing- Original draft preparation.

Nozomi Kubota: Interpretation of data, Writing-Review & Editing.

Masayoshi Shiota: Interpretation of data, Writing-Review & Editing, Supervision.

### Approval by Institutional Review Board (IRB)

The study protocol was approved by the Ethics Committee of the Kawakita General Hospital (Approval no. 2022-0022).

## References

[1] Aygun S, Tokgozoglu L. Comparison of current international guidelines for the management of dyslipidemia. J Clin Med. 2022;11(23):7249. 36498823 10.3390/jcm11237249 PMC9737468

[2] Kinoshita M, Yokote K, Arai H, et al. Japan Atherosclerosis Society (JAS) guidelines for prevention of atherosclerotic cardiovascular diseases 2017. J Atheroscler Thromb. 2018;25(9):846-984. 30135334 10.5551/jat.GL2017 PMC6143773

[3] Mauvais-Jarvis F, Bairey Merz N, Barnes PJ, et al. Sex and gender: modifiers of health, disease, and medicine. Lancet. 2020;396(10250):565-82. 32828189 10.1016/S0140-6736(20)31561-0 PMC7440877

[4] Gouni-Berthold I, Berthold HK, Mantzoros CS, et al. Sex disparities in the treatment and control of cardiovascular risk factors in type 2 diabetes. Diabetes Care. 2008;31(7):1389-91. 18375411 10.2337/dc08-0194 PMC2453666

[5] Xia S, Du X, Guo L, et al. Sex differences in primary and secondary prevention of cardiovascular disease in China. Circulation. 2020;141(7):530-9. 32065775 10.1161/CIRCULATIONAHA.119.043731

[6] Lee CMY, Mnatzaganian G, Woodward M, et al. Sex disparities in the management of coronary heart disease in general practices in Australia. Heart. 2019;105(24):1898-904. 31337667 10.1136/heartjnl-2019-315134

[7] Rachamin Y, Grischott T, Rosemann T, et al. Inferior control of low-density lipoprotein cholesterol in women is the primary sex difference in modifiable cardiovascular risk: a large-scale, cross-sectional study in primary care. Atherosclerosis. 2021;324:141-7. 33810858 10.1016/j.atherosclerosis.2021.02.024

[8] Nguyen TV, Tran DTT, Ngo TTK, et al. Sex difference in control of low-density lipoprotein cholesterol in older patients after acute coronary syndrome. Geriatrics. 2022;7(4):71. 35893318 10.3390/geriatrics7040071 PMC9326734

[9] Gamboa CM, Colantonio LD, Brown TM, et al. Race-sex differences in statin use and low-density lipoprotein cholesterol control among people with diabetes mellitus in the reasons for geographic and racial differences in stroke study. J Am Heart Assoc. 2017;6(5):e004264. 28490523 10.1161/JAHA.116.004264 PMC5524054

[10] Nanna MG, Wang TY, Xiang Q, et al. Sex differences in the use of statins in community practice. Circ Cardiovasc Qual Outcomes. 2019;12(8):e005562. 31416347 10.1161/CIRCOUTCOMES.118.005562 PMC6903404

[11] Miya A, Nakamura A, Suzuki Y, et al. Frequency and determinants of lipid management target achievement in primary prevention of cardiovascular disease in type 2 diabetes. Diabetol Int. 2024;15(3):465-73. 39101195 10.1007/s13340-024-00712-x PMC11291843

[12] Schmittdiel JA, Traylor A, Uratsu CS, et al. The association of patient-physician gender concordance with cardiovascular disease risk factor control and treatment in diabetes. J Womens Health. 2009;18(12):2065-70. 10.1089/jwh.2009.1406 PMC2828159 20044871

[13] Lau ES, Hayes SN, Volgman AS, et al. Does patient-physician gender concordance influence patient perceptions or outcomes. J Am Coll Cardiol. 2021;77(8):1135-8. 33632488 10.1016/j.jacc.2020.12.031

[14] Ye H, Yi J. Patient-physician race concordance, physician decisions, and patient outcomes. Rev Econ Stat. 2023;105(4):766-79.

[15] Chekijian S, Kinsman J, Taylor RA, et al. Association between patient-physician gender concordance and patient experience scores. Is there gender bias. Am J Emerg Med. 2021;45:476-82. 33069544 10.1016/j.ajem.2020.09.090

[16] Greenwood BN, Carnahan S, Huang L. Patient-physician gender concordance and increased mortality among female heart attack patients. Proc Natl Acad Sci U S A. 2018;115(34):8569-74. 30082406 10.1073/pnas.1800097115 PMC6112736

[17] Baker R, Freeman GK, Haggerty JL, et al. Primary medical care continuity and patient mortality: a systematic review. Br J Gen Pract. 2020;70(698):e600-11. 32784220 10.3399/bjgp20X712289 PMC7425204

[18] Canadian Institute for Health Information. Continuity of care with family medicine physicians: why it matters [Internet]. 2024 [cited 2024 Oct 9]. Available from: https://secure.cihi.ca/free_products/UPC_ReportFINAL_EN.pdf.

[19] Zou G. A modified poisson regression approach to prospective studies with binary data. Am J Epidemiol. 2004;159(7):702-6. 15033648 10.1093/aje/kwh090

[20] Barros AJD, Hirakata VN. Alternatives for logistic regression in cross-sectional studies: an empirical comparison of models that directly estimate the prevalence ratio. BMC Med Res Methodol. 2003;3(1):21. 14567763 10.1186/1471-2288-3-21 PMC521200

[21] Sandhu H, Adams A, Singleton L, et al. The impact of gender dyads on doctor-patient communication: a systematic review. Patient Educ Couns. 2009;76(3):348-55. 19647969 10.1016/j.pec.2009.07.010

[22] Dagostini CM, Bicca YA, Ramos MB, et al. Patients’ preferences regarding physicians’ gender: a clinical center cross-sectional study. Sao Paulo Med J. 2022;140(1):134-43. 35043868 10.1590/1516-3180.2021.0171.R1.08062021 PMC9623840

[23] Konnyu KJ, Yogasingam S, Lépine J, et al. Quality improvement strategies for diabetes care: effects on outcomes for adults living with diabetes. Cochrane Database Syst Rev. 2023;5(5):CD014513. 37254718 10.1002/14651858.CD014513 PMC10233616

[24] Chapman EN, Kaatz A, Carnes M. Physicians and implicit bias: how doctors may unwittingly perpetuate health care disparities. J Gen Intern Med. 2013;28(11):1504-10. 23576243 10.1007/s11606-013-2441-1 PMC3797360

[25] FitzGerald C, Hurst S. Implicit bias in healthcare professionals: a systematic review. BMC Med Ethics. 2017;18(1):19. 28249596 10.1186/s12910-017-0179-8 PMC5333436

[26] Champagne-Langabeer T, Hedges AL. Physician gender as a source of implicit bias affecting clinical decision-making processes: a scoping review. BMC Med Educ. 2021;21(1):171. 33740973 10.1186/s12909-021-02601-2 PMC7980423

[27] Jenkins CR, Chapman KR, Donohue JF, et al. Improving the management of COPD in women. Chest. 2017;151(3):686-96. 27816445 10.1016/j.chest.2016.10.031

[28] Pelletier R, Ditto B, Pilote L. A composite measure of gender and its association with risk factors in patients with premature acute coronary syndrome. Psychosom Med. 2015;77(5):517-26. 25984818 10.1097/PSY.0000000000000186

[29] Butkus R, Serchen J, Moyer DV, et al. Achieving gender equity in physician compensation and career advancement: a position paper of the American College of Physicians. Ann Intern Med. 2018;168(10):721-3. 29710100 10.7326/M17-3438

[30] Cohen DJ, Balasubramanian BA, Gordon L, et al. A national evaluation of a dissemination and implementation initiative to enhance primary care practice capacity and improve cardiovascular disease care: the ESCALATES study protocol. Implement Sci. 2016;11(1):86. 27358078 10.1186/s13012-016-0449-8 PMC4928346

[31] Kuzel AJ, Cuellar A, Nichols L. The EvidenceNOW practice support initiative: the heart of Virginia healthcare. J Am Board Fam Med. 2022;35(5):979-89. 36257695 10.3122/jabfm.2022.05.210021

[32] Balasubramanian BA, Lindner S, Marino M, et al. Improving delivery of cardiovascular disease preventive services in small-to-medium primary care practices. J Am Board Fam Med. 2022;35(5):968-78. 10.3122/jabfm.2022.AP.220038 36096660

[33] B Sussman J, Holleman RG, Youles B, et al. Quality improvement and personalization for statins: the quips quality improvement randomized trial of veterans’ primary care statin use. J Gen Intern Med. 2018;33(12):2132-7. 30284172 10.1007/s11606-018-4681-6 PMC6258630

[34] Mitani H, Suzuki K, Ako J, et al. Achievement rates for low-density lipoprotein cholesterol goals in patients at high risk of atherosclerotic cardiovascular disease in a real-world setting in Japan. J Atheroscler Thromb. 2023;30(11):1622-34. 36928267 10.5551/jat.63940 PMC10627744

[35] Jiang P. Legislating for transgender people: a comparative study of the change of legal gender in Hong Kong, Singapore, Japan and the United Kingdom [Internet]. 2013 [cited 2024 Jul 9]. Available from: https://www.academia.edu/4171417/Legislating_for_Transgender_People_A_Comparative_Study_of_the_Change_of_Legal_Gender_in_Hong_Kong_Singapore_Japan_and_the_United_Kingdom

